# Correlation of Liver and Myocardium Iron Concentration Determined by Magnetic Resonance Imaging With Serum Ferritin in Non-Transfusion-Dependent Thalassemia Patients

**DOI:** 10.7759/cureus.27467

**Published:** 2022-07-29

**Authors:** Nagenthran Gayathri, M Vasantha Kumar, Thangam Vinoth, Radhan Prabhu, S Krishnabharath

**Affiliations:** 1 Radiodiagnosis, Medal Diagnostics Center, Chennai, IND; 2 Radiology, ACS Medical College and Hospital, Chennai, IND; 3 Radiodiagnosis, ACS Medical College and Hospital, Chennai, IND; 4 Radiodiagnosis, MGM Health Care, Chennai, IND; 5 Surgical Gastroenterology, Tirunelveli Medical College and Hospital, Tirunelveli, IND

**Keywords:** non-transfusion-dependent thalassemia, correlation, myocardium iron concentration, serum ferritin, mri

## Abstract

Background

The primary factor associated with fatality in thalassemia patients is heavy cardiac complications. Currently, magnetic resonance imaging (MRI) is accepted as the non-invasive modality of choice for diagnosing iron overload in the liver. This study aimed to correlate liver iron concentration (LIC) and myocardium iron concentration (MIC) determined by MRI and clinical and biochemical parameters in non-transfusion-dependent thalassemia (NTDT) patients.

Methodology

This prospective study was conducted in the radiology department from October 2016 to September 2018. A total of 30 patients were included. Using Siemens MAGNETOM^®^ Avanto 1.5T, iron was quantified with a body matrix coil. Sequences performed were gradient-echo 8 and 12 for the myocardium and liver, respectively. Dual-echo fast spoiled gradient-echo in/out phase and diffusion-weighted images were used. Iron values were calculated using T2* spreadsheet analysis software version 3.1. Data were analyzed using coGuide software V.1.03.

Results

The mean age of the participants was 24.9 ± 12.6 years. There was a very strong positive correlation between LIC and serum ferritin. There was a strong negative correlation between LIC and hemoglobin. Between LIC and MIC, there was a marginally favorable relationship (r_s_ value = 0.077, p-value = 0.985).

Conclusions

When MRI is not available, serum ferritin can be used as an alternative to diagnose iron overload in patients with NTDT.

## Introduction

Thalassemia is a major heterogeneous group of inherited blood disorders caused by insufficient or non-functional hemoglobin [1,2]. It is an autosomal recessive disorder that can be transferred to the offspring when both parents are either affected or are carriers. The mutations or deletions in the Hb genes result in the absence or underproduction of alpha or beta chains, and there exist about 200 mutations majorly responsible for causing thalassemias [3]. Cardiac issues, such as heart failure and arrhythmia, are the main cause of death in people with thalassemia, accounting for up to 71% of all fatalities. Additionally, 30% of patients have liver fibrosis caused by iron excess [4,5]. If thalassemia is not treated, it can be fatal. In the past years, it has emerged as a public health concern throughout the Middle East, Mediterranean, and Southeast Asian regions [6]. It is also a significant cause of morbidity and mortality among the Indian population [7,8]. Depending on the requirement for blood transfusions, two terminologies, namely, transfusion-dependent (TDT) and non-transfusion-dependent thalassemia (NTDT), are commonly used in clinical settings [9-11].

Patients suffering from NTDT require transfusions occasionally at fixed time intervals. There is no need for regular or lifelong transfusions for survival. This type of thalassemia encompasses three clinically distinct forms, namely, β-thalassemia intermedia, hemoglobin E/β-thalassemia (mild or moderate) (Hb E), and α-thalassemia intermedia, also known as hemoglobin H (Hb H) disease [12,13]. The transfusion requirements of an NTDT patient can change over time, and there is considerable variability in the transfusion needs of different individuals [14]. Iron overload caused by increased intestinal iron absorption is a serious clinical problem in NTDT patients, especially as they age. In persons with NTDT, iron overload causes a range of clinical ramifications. They include liver fibrosis or cirrhosis, increased risk of hepatocellular carcinoma, thrombosis, bone disease, pulmonary hypertension, cerebrovascular and neural damage, and endocrinopathy [15,16]. Given its safety and reproducibility, magnetic resonance imaging (MRI) employing R2 or T2* methods has replaced liver biopsy as the gold standard for assessing liver iron concentration (LIC) [17]. The T2* method of MRI is also being utilized to measure the myocardium iron concentration (MIC) in milliseconds. As MIC increases, T2* becomes shorter. The relative unavailability and higher cost of MRI technology, particularly in regions where the condition is most widespread, continue to be one of the major obstacles to its use for quantifying iron overload. Several nations in the developing world are included in those regions, including sub-Saharan Africa, the Mediterranean region, the Middle East, India, and Southeast Asia. This has led to the discovery of serum ferritin levels in TDT and NTDT that are correlated with hepatic iron excess [18]. An earlier study found that the relationship between serum ferritin and cardiac T2* is not as strong as the relationship between serum ferritin and LIC [19]. However, T2* MRI is expensive, not widely available, and its interpretation needs an expert radiologist, which limits its application. Hence, this study was conducted to correlate LIC and MIC determined by MRI with clinical and biochemical parameters in NTDT patients.

## Materials and methods

This prospective study was conducted in the radiology department from October 2016 to September 2018. NTDT patients attending the departmental outpatient department (OPD) during the study period were included using a convenient sampling method. Study approval was obtained from the Institutional Ethical Committee (reference number: CSP-MED/16/OCT/31/162), and written informed consent was obtained from all participants before enrolling them.

A total of 30 patients aged between five and 50 years after being diagnosed with NTDT were included in the study. We excluded NTDT patients on chelation therapy, those with fever or active infections (as their blood tests interfered with serum ferritin levels), and those with contraindications to MRI. Serum ferritin levels were obtained using the chemiluminescent method.

Iron quantification was done using Siemens MAGNETOM® Avanto 1.5T with a body matrix coil. Sequences performed included gradient-echo (GRE) 8 and 12 for myocardium and liver, respectively. Dual-echo fast spoiled gradient-echo (FSPGR) in/out phase and diffusion-weighted image were also utilized. Iron values were calculated using T2* spreadsheet analysis. The region of interest (ROI) was 2.5 cm^2^ ± 5 mm^2^ and 15 cm^2^ ± 5 mm^2^ in the interventricular septum and peripheral of the liver (devoid of blood vessels), respectively. The ROI was copied across all sets of GRE 8 and 12 echoes for the myocardium and liver, respectively. Each image generated a pair of numbers TE and mean signal intensity (SI) of ROI. It was transferred to the T2* analysis spreadsheet version 3.1 to determine T2*, R2*, MIC [20], and LIC [21].

Blood samples of all participants were collected using phlebotomy and were tested for serum ferritin using the chemiluminescence technique.

Statistical analysis

The severity of LIC and MIC was considered as primary outcome variables. The diagnosis, MRI, and serum ferritin were considered the primary explanatory variables. Descriptive statistics were used to analyze data following the study’s objectives. Data were expressed as the mean, 95% confidence interval (CI; lower and upper bounds), minimum and maximum, and percentage, where appropriate. The count variables were analyzed using the chi-square test expressed as a number. The data were depicted in a scatter plot, and the correlation between various parameters was determined using the Spearman rank correlation coefficient (rs). P-values lower than 0.05 were regarded as statistically significant. CoGuide software (version 1.03) was used for data analysis [22].

## Results

A total of 30 patients were included in the final analysis. The mean age was 24.9 ± 12.6 years (ranging from six to 48 years). In total, 17 (56.67%) were male patients, and 13 (43.33%) were female patients. In our study, 15 (50%) patients were diagnosed with beta-thalassemia Intermedia, nine (30%) were diagnosed with Hb E, and six (20%) were diagnosed with Hb H. Regarding the severity of LIC overload, six (20%) had normal LIC, 18 (60%) had a mild overload, and six (20%) had a moderately severe overload. The mean LIC was 4.63 ± 2.38 µg/g, ranging from 1.20 to 9.50 µg/g. MIC was normal in 29 (96.67%) patients, and one (3.33%) patient had mild MIC. The mean MIC was 0.73 ± 0.24 mg/g, ranging from 0.43 to 1.46 mg/g. The mean ferritin was 500.81 ± 448.44 ng/mL, ranging from 100.50 to 2,014 ng/mL. The mean hemoglobin was 8.23 ± 1 g/dL, ranging from 6.40 to 9.80 g/dL. The mean liver size was 14.88 ± 2.09 cm, ranging from 11.30 to 18 cm. The mean spleen size was 13.59 ± 2.1 cm, ranging from 8.50 to 16.70 cm. Only one (3.33%) patient had undergone transfusion six years back (Table 1).

**Table 1:** Summary of baseline parameters (N = 30). LIC: liver iron concentration; MIC: myocardium iron concentration; Hb E: hemoglobin E; Hb H: hemoglobin H

Parameter	Summary
Age (in years)	24.9 ± 12.6 (range = 6–48)
Gender	
Male	17 (56.67%)
Female	13 (43.33%)
Diagnosis	
Beta-thalassemia Intermedia	15 (50%)
Hb E	9 (30%)
Hb H	6 (20%)
Severity of LIC	
Normal	6 (20.00%)
Mild	18 (60.00%)
Moderate	6 (20.00%)
LIC (in µg/g)	4.63 ± 2.38 (range = 1.20–9.50)
MIC	
Normal	29 (96.67%)
Mild	1 (3.33%)
MIC (in mg/g)	0.73 ± 0.24 (range = 0.43–1.46)
Ferritin (in ng/mL)	500.81 ± 448.44 (range = 100.50–2014)
Hemoglobin (in g/dL)	8.23 ± 1 (range = 6.40–9.80)
Liver size (in cm)	14.88 ± 2.09 (range = 11.30–18)
Spleen size (in cm)	13.59 ± 2.1 (range = 8.50–16.70)
Transfusion (six years back)	1 (3.33%)

Of the 15 patients diagnosed as intermedia, two (13.33%) had a normal, nine (60%) had mild, and four (26.67%) had a moderate iron overload. Of the nine patients diagnosed as Hb E subtypes, three (33.33%) had normal, five (5.56%) had mild, and one (11.11%) had a moderate iron overload. Of the six patients diagnosed as Hb H subtypes, one (16.67%) had normal and four (66.67%) had a mild iron overload. The difference in the proportion of diagnosis across severity was statistically not significant (p-value = 0.743) (Table 2).

**Table 2:** Comparison of diagnosis across severity (N = 30). LIC: liver iron concentration; Hb E: hemoglobin E; Hb H: hemoglobin H

Diagnosis	Severity of LIC			Chi-square	P-value
	Normal	Mild	Moderate		
Intermedia (N = 15)	2 (13.33%)	9 (60%)	4 (26.67%)	1.963	0.743
Hb E (N = 9)	3 (33.33%)	5 (55.56%)	1 (11.11%)		
Hb H (N = 6)	1 (16.67%)	4 (66.67%)	1 (16.67%)		

**Figure 1:**
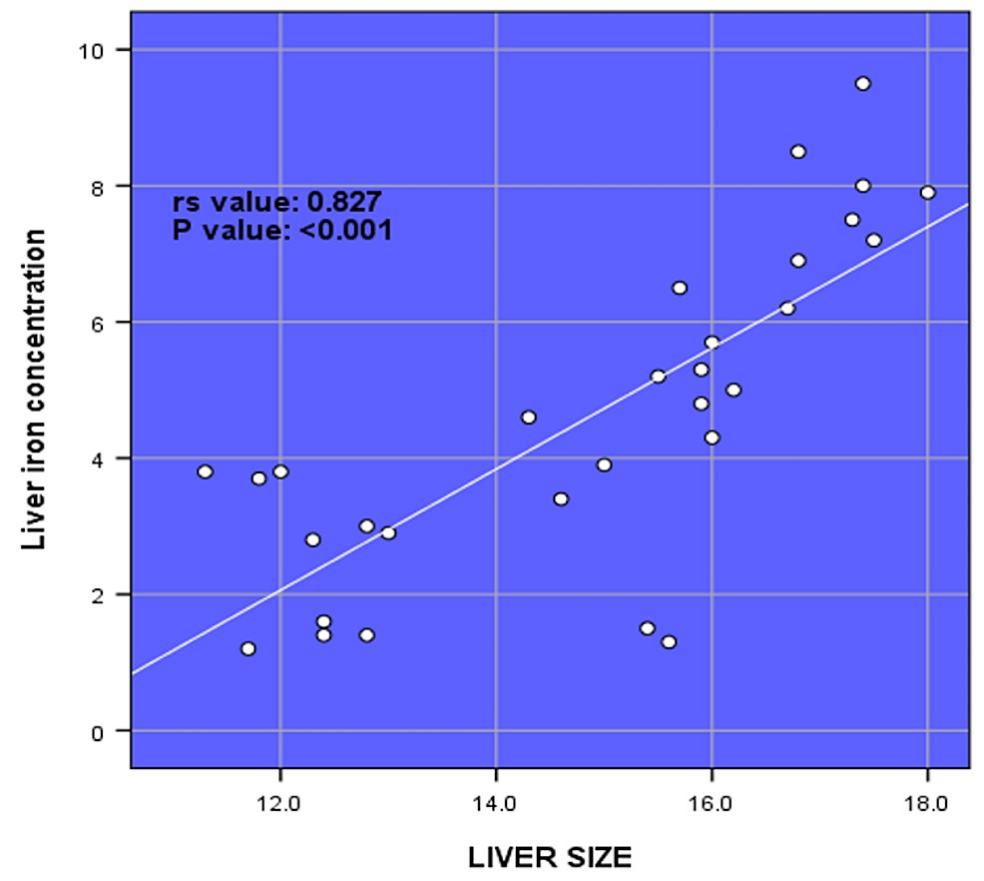
Spearman rank correlation (rs) between liver iron concentration and liver size (N = 30).

**Figure 2:**
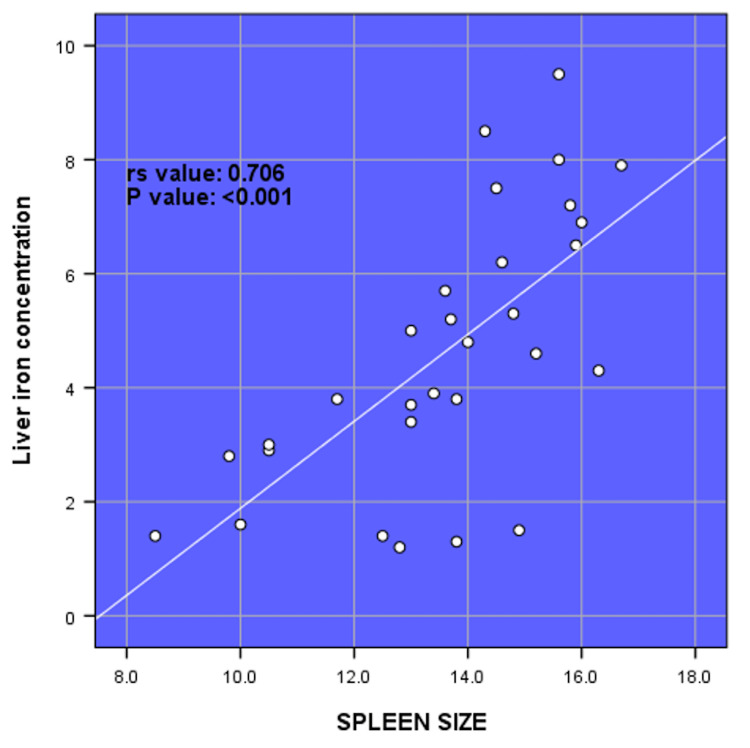
Spearman rank correlation (rs) between liver iron concentration and liver size (N = 30).

**Figure 3:**
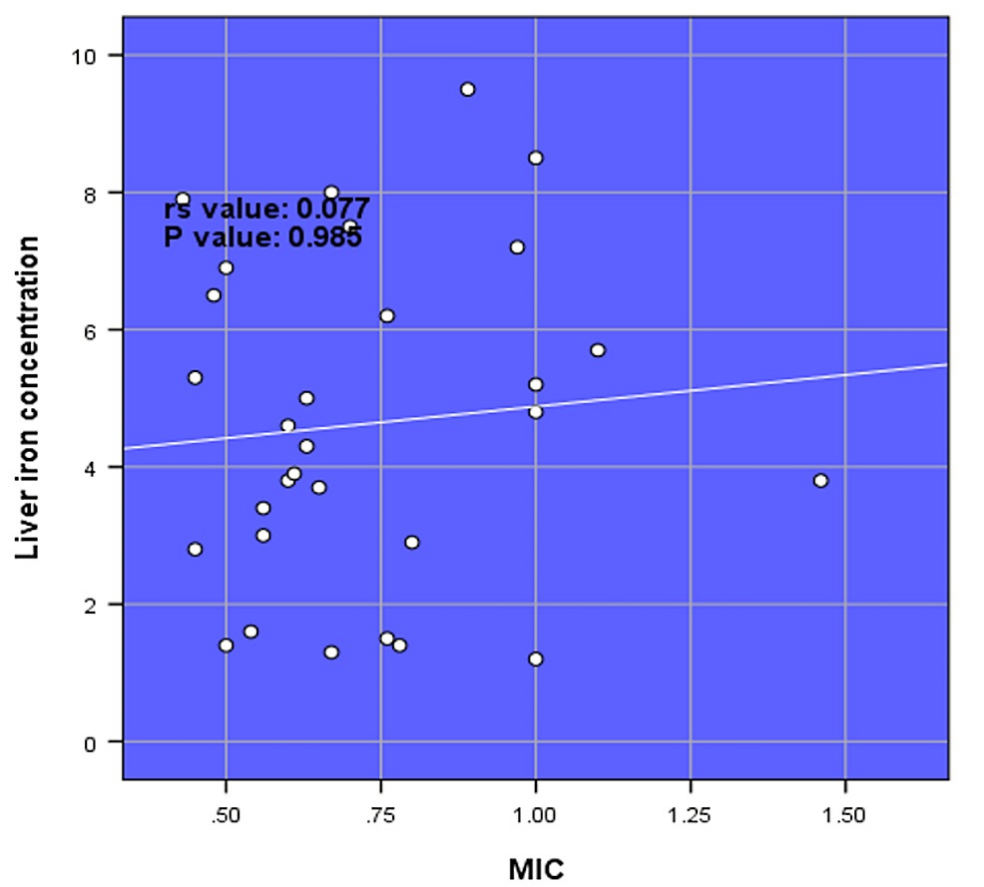
Spearman rank correlation (rs) between liver iron concentration and myocardium iron concentration (N = 30).

There was a very strong positive correlation between LIC and serum ferritin (rs value = 0.995, p-value = <0.001). There was a strong negative correlation between LIC and hemoglobin (rs value = -0.812, p-value = <0.001). There was a strong positive correlation between LIC and liver size (rs value = 0.827, p-value = <0.001) (Figure 1). There was a strong positive correlation between LIC and spleen size (rs value = 0.706, p-value = <0.001) (Figure 2). There was no correlation between LIC and MIC (rs value = 0.077, p-value = 0.985) (Figure 3).

## Discussion

The goal of this investigation was to determine if heart and hepatic iron loads in individuals with or without thalassemia could be detected using blood ferritin levels. The age group of our study population was 6-48 years. Hemoglobin ranged from 6.40 to 9.80 g/dL, and only one (3.33%) patient had undergone transfusion six years back. Tantiworawit et al. reported that the clinical risk factors for hepatic iron overload were age >20 years and cumulative red blood cell transfusion of >10 units [23]. In our study, the majority (50%) of the study population was diagnosed with beta-thalassemia intermedia. According to a study by Weatherall et al., beta-thalassemia intermedia is globally one of the most prevalent forms of NTDT, affecting about 70,000 children annually [13]. Musallam et al. reported that the beta-thalassemia intermedia phenotype shows a wide spectrum of disease severity [24].

A study conducted in Shiraz, Iran, reported that serum ferritin levels show a statistically significant positive correlation with LIC (rs = 0.718, p = 0.001), which was consistent with the very strong positive correlation between LIC and serum ferritin found in this study (rs = 0.995, p = 0.001) [25]. Hepatic T2* levels and serum ferritin levels had a somewhat positive correlation according to Zamani et al. [26]. Similarly, in the study by Chuansumrit et al., a significant correlation between liver T2* and serum ferritin were expressed as the equation: T2* (ms) = 28.080-7.629 log ferritin (μg/L) (r^2^ = 0.424, p = <0.001) [27]. Our results are consistent with those reported by Tantiworawit et al., who observed that the correlation coefficient between serum ferritin and LIC was 0.60 (p < 0.001) [23]. Karimi et al. observed that serum ferritin levels showed a statistically significant negative correlation with T2 hepatic MRI (r = −0.290, p = 0.003) and a positive correlation with LIC (r = 0.426, p = <0.001) in patients with beta-thalassemia intermedia [28]. In this study, we found no significant correlation between LIC and MIC (rs = 0.077, p = 0.985). Serum ferritin level was significantly correlated with LIC (rs = 0.65, p < 0.001) and hepatic T2* value (rs = -0.62, p < 0.001), but not with cardiac T2* value (rs  = -0.20, p = 0.07). In a cross-sectional study, a weak negative non-significant correlation was reported between hepatic and cardiac T2* values (p = 0.4, r = -0.37), and a weakly positive correlation was found for cardiac T2* (p = 0.04, r = -0.37) [29]. Wahidiyat et al. reported a significant but weak correlation between cardiac T2* MRI and serum ferritin, and a slightly more significant correlation between LIC and serum ferritin [30]. In this study, we found a strong positive correlation between LIC and liver size. This shows that with the increase in LIC, the size of the liver increases. It was also observed that there was a strong positive correlation between LIC and spleen size. The size of the spleen increased with the increase in LIC.

In addition to assessing the usefulness of serum ferritin over or as an alternative to MRI in estimating liver and myocardium iron overload, the only limitation of the current study was its small sample size which was resolved by performing appropriate statistical analyses.

## Conclusions

Our study concludes using serum ferritin as an indicator in estimating LIC and MIC because a positive correlation was obtained upon calculating the Spearman rank correlation coefficient. Hence, we encourage the use of serum ferritin in diagnosing the same when MRI is unavailable or is unaffordable for the patients. Because our study is limited by its smaller sample size, studies with larger sample sizes are encouraged.
